# Optimization and characterization of spray-dried IgG formulations: a design of experiment approach

**DOI:** 10.1186/s40199-017-0187-8

**Published:** 2017-10-24

**Authors:** Homa Faghihi, Abdolhosein Rouholamini Najafabadi, Alireza Vatanara

**Affiliations:** Department of Pharmaceutics, Faculty of Pharmacy, Tehran University of Medical Sciences, Tehran, Iran

**Keywords:** Spray-drying, IgG, Molecular stability, Experimental design, Box-Behnken, Aerosol delivery

## Abstract

**Background:**

The purpose of the present study is to optimize a spray-dried formulation as a model antibody regarding stability and aerodynamic property for further aerosol therapy of this group of macromolecules.

**Method:**

A three-factor, three-level, Box-Behnken design was employed milligrams of Cysteine (X_1_), Trehalose (X_2_), and Tween 20 (X_3_) as independent variables. The dependent variables were quantified and the optimized formulation was prepared accordingly. SEC-HPLC and FTIR-spectroscopy were conducted to evaluate the molecular and structural status of spray-dried preparations. Particle characterization of optimized sample was performed with the aid of DSC, SEM, and TSI examinations.

**Results:**

Experimental responses of a total of 17 formulations resulted in yield values, (Y_1_), ranging from 21.1 ± 0.2 to 40.2 ± 0.1 (%); beta-sheet content, (Y_2_), from 66.22 ± 0.19 to 73.78 ± 0.26 (%); amount of aggregation following process, (Y_3_), ranging from 0.11 ± 0.03 to 0.95 ± 0.03 (%); and amount of aggregation upon storage, (Y_4_), from 0.81 ± 0.01 to 3.13 ± 0.64 (%) as dependent variables. Results—except for those of the beta sheet content—were fitted to quadratic models describing the inherent relationship between main factors.

**Conclusion:**

Co-application of Cysteine and Tween 20 preserved antibody molecules from molecular degradation and improved immediate and accelerated stability of spry-dried antibodies. Validation of the optimization study indicated high degree of prognostic ability of response surface methodology in preparation of stable spray-dried IgG.

**Graphical abstract:**

**Graphical abstract:**
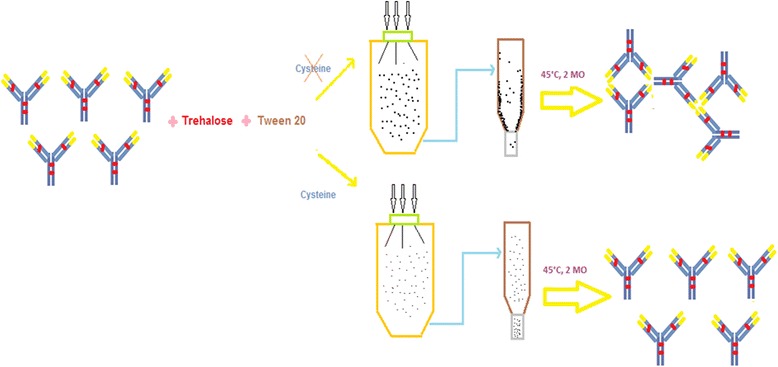
Spray drying of IgG in the presence of Trehalose, Cysteine and Tween 20.

## Background

Antibody-based drugs are regarded as major influential components in the treatment of cancers, autoimmune, and inflammatory diseases [1]. Considering about 50 approved monoclonal antibodies, Omalizumab, Bevacizumab, Palivizumab, and Cetuximab are administered in respiratory diseases and more than 9 molecules are at different stages of clinical trials [2].

Spray-drying is an emerging technology for the processing of antibody dry powders [3]. However, pure antibody solutions have been shown to become substantially aggregated during this process [4, 5]. Such destabilization is rationally attributed to the shearing stress in the nozzle, thermal stress within drying, and surface adsorption of protein at the air-liquid interfaces during atomization [6]. Incorporation of appropriate excipients is therefore critical for preserving protein stability.

Trehalose and Tween 20 are applied as excipients in preventing proteins against destabilization during the spray-drying process [7]. Trehalose is one of the most promising inhibitors of antibody aggregation with high glass transition temperature, low hygroscopicity, and strong water replacement efficacy [8]. Surfactants such as Tween 20 have also been shown to occupy the air–liquid interface in competition with protein molecules, thus avoiding subsequent protein unfolding and aggregation [9, 10].

Although polysorbates were repeatedly shown to stabilize various proteins against surface denaturation within spray-drying, auto-xidation of poly-oxy ethylene groups at high temperatures is considered as a major challenge in Tween-containing formulations after storage [11, 12].

Our previous investigation introduced Cysteine as an appropriate excipient in IgG formulation regarding antibody stability as well as its aerodynamic behavior [13]. In the current study, the combination of Cysteine, Trehalose, and Tween 20 was applied to not only enhance the molecular and thermodynamic stability of IgG, but also to examine whether the Cysteine—as an anti-oxidant—could protect Tween 20 from auto-oxidation following storage.

One particular feature of this study is a statistical comparison between Cysteine, Trehalose, and Tween 20 as stabilizing agents for the spray-dried IgG formulation. The other objective was to probe the existing interactions between immunoglobulin G and sugar, and amino acid and surfactant. To achieve this purpose, a Box-Behnken experimental design was applied to optimize the best combination of the aforementioned additives in spray-dried IgG formulation as a model antibody. The evaluated responses were yield of process, beta sheet content of antibody, and amount of induced aggregation following process and upon storage. Subsequently, optimized formulation was characterized in terms of surface morphology and amorphous/crystalline pattern for further aerosol delivery.

## Methods

### Materials

L-Cysteine, Tween 20, Phosphoric acid, and KBr were purchased from Sigma (Germany); Trehalose dihydrate, Sodium sulfate, and Disodium hydrogen phosphate were provided by Merck (Germany); Human IgG with molecular weight of about 150 kDa was supplied by Kedrion (Italy); antibody solution was dialyzed with deionized water (bag cut off: 8 KDa).

### Box-Behnken experimental design

A three-factor, three-level Box-Behnken design was employed for the optimization process using the statistical software Design Expert 6.0.10 (Stat-Ease Inc., USA). The non-linear quadratic model generated by the design was:

1 $$ \mathrm{Yi}={\mathrm{b}}_0+{\mathrm{b}}_1{\mathrm{X}}_1+{\mathrm{b}}_2{\mathrm{X}}_2+{\mathrm{b}}_3{\mathrm{X}}_3+{\mathrm{b}}_{12}{\mathrm{X}}_1{\mathrm{X}}_2+{\mathrm{b}}_{13}{\mathrm{X}}_1{\mathrm{X}}_3+{\mathrm{b}}_{23}{\mathrm{X}}_2{\mathrm{X}}_3+{\mathrm{b}}_{11}{{\mathrm{X}}_1}^2+{\mathrm{b}}_{22}{{\mathrm{X}}_2}^2+{\mathrm{b}}_{33}{{\mathrm{X}}_3}^2 $$

In which Y_i_ is the measured response of each dependent variable; b_0_ is the intercept; b_1_ to b_33_ are the regression coefficients of the factors; and X_1_, X_2_, and X_3_ are the coded levels of independent variables. The term X_1_ X_2_, X_1_ X_3,_ X_2_ X_3_ and X_i_^2^ (*i* = 1, 2, or 3) exhibit the interaction and the quadratic terms respectively. A description of the independent and dependent variables is given in Table 1. The models were evaluated in terms of statistically significant *p*-value and CE.

**Table 1:** Variables in Box Behnken design

Factor	Levels used		
Independent variables	−1	0	1
X1 = Cysteine (%*w*/w)	25	37.5	50
X2 = Trehalose (%w/w)	60	105	150
X3 = Tween 20 (%w/w)	0	0.03	0.05
Dependent variables			Constraints
Y1 = Yield (%)			Maximize
Y2 = Beta- sheet content (%)			66 ≤Y2≤73
Y3 = Amount of aggregation immediately following spray drying (%)			Minimize
Y4 = Amount of aggregation following 2 month storage at 45 °C (%)			Minimize

### Preparation of antibody formulations and spray-drying

Primary dialyzed solutions of IgG had an antibody concentration of 100 mg/ml. All formulations containing Cysteine and Trehalose with or without Tween 20 were prepared according to the experimental design in Table 2 and immediately spray-dried using a lab scale spray dryer (Buchi 191, Switzerland). Spray-drying conditions were as follows: inlet temperature 100 °C, airflow rate 600 Nl/h, aspirator setting 100%, outlet temperature 56–60 °C, and feeding rate 10% (1.8 ml/min). The collected powders were subsequently stored in glass vials at 4 °C for further examinations.

**Table 2:** Composition and observed responses in Box–Behnken design

Batch	Independent variables			Dependent variables			
	X1 (%)	X2 (%)	X3 (%)	Y1(%)	Y2(%)	Y3(%)	Y4(%)
1	0	0	0	38.3 ± 0.2	69.21 ± 0.32	0.11 ± 0.05	0.81 ± 0.01
2	0	1	1	35.1 ± 0.1	72.32 ± 0.24	0.32 ± 0.02	1.22 ± 0.12
3	0	0	0	40.2 ± 0.1	70.11 ± 0.11	0.11 ± 0.03	0.91 ± 0.08
4	0	0	0	36.3 ± 0.3	69.21 ± 0.54	0.23 ± 0.09	0.87 ± 0.03
5	0	− 1	1	33.4 ± 0.2	67.43 ± 0.42	0.65 ± 0.04	2.16 ± 0.23
6	0	0	0	37.1 ± 0.1	70.09 ± 0.27	0.12 ± 0.01	0.83 ± 0.07
7	−1	− 1	0	21.1 ± 0.2	66.22 ± 0.19	0.83 ± 0.08	2.68 ± 0.42
8	0	0	0	35.2 ± 0.1	71.34 ± 0.12	0.15 ± 0.03	0.95 ± 0.05
9	−1	1	0	24.1 ± 0.3	72.29 ± 0.82	0.47 ± 0.02	1.52 ± 0.11
10	1	1	0	33.7 ± 0.5	73.44 ± 0.47	0.29 ± 0.02	0.94 ± 0.04
11	0	1	−1	37.5 ± 0.1	73.78 ± 0.26	0.51 ± 0.05	1.82 ± 0.19
12	1	0	1	35.2 ± 0.7	71.35 ± 0.18	0.13 ± 0.01	0.86 ± 0.07
13	-1	0	-1	30.1 ± 0.5	69.28 ± 0.43	0.56 ± 0.07	1.98 ± 0.42
14	-1	0	1	25.2 ± 0.8	69.42 ± 0.35	0.42 ± 0.05	1.78 ± 0.09
15	0	-1	-1	30.6 ± 0.2	66.37 ± 0.93	0.95 ± 0.03	3.13 ± 0.64
16	1	0	-1	32.2 ± 0.8	71.92 ± 0.22	0.46 ± 0.01	1.64 ± 0.29
17	1	-1	0	22.8 ± 0.9	66.56 ± 0.39	0.53 ± 0.05	2.13 ± 0.56

### Size exclusion chromatography

IgG-containing samples were analyzed for quantification of aggregates/fragments by SEC-HPLC. A TSK 3000 SWXL column (Tosoh, Biosep, Germany) was applied. The running buffer was composed of 0.1 M Disodium hydrogen phosphate dihydrate and 0.1 M Sodium sulfate, and pH 6.8. The flow rate of pump (Jasco, USA) was set to 0.5 mL/min and the injection volume to 20 μL. Each weighed sample containing 2.5 mg of IgG was dissolved in 1 ml of deionized water and mixed. Then the solution was filtered through 0.45 μ syringe filters before analysis. Antibody aggregates, monomers, and fragments were determined using a UV detector (Jasco, USA) at 280 nm. For each sample, analysis was performed in triplicate.

### Fourier transform infrared spectroscopy (FTIR)

FTIR spectroscopy was used to investigate the conformational stability of the spray-dried IgG formulations in the solid state. IR measurements were performed using a FTIR spectrometer (Nicolet Magna, USA) at room temperature. Samples were prepared by mixing about 2 mg of spray-dried powder with 200 mg KBr and a 6–7 T of pressure to prepare a compact tablet. The Jasco Spectra Manager® software was applied to analyze the changes in the secondary structure of IgG in the amide I region (1600–1700 cm^−1^) of the spectrum. Curve fitting was carried out through application of a mixed Gaussian/Lorentzian function.

### Evaluation of storage stability

The storage stability of spray-dried samples was evaluated following incubation for 2 months at 45 °C and RH of 60%. Since the formation of soluble aggregates was previously reported to be the most prevalent degradation pathway of IgG molecules upon storage conditions, each sample was reconstituted in water at a 2.5 mg/ml concentration and analyzed using SEC-HPLC. The analysis was performed similar to section 2.4 with same buffer system and procedure.

### Differential scanning Calorimetry (DSC)

The thermal behavior of the powders was considered when applying a differential scanning calorimeter (Mettler Toledo, Switzerland). Four samples were prepared and DSC was done once per sample. The equipment was calibrated with indium and zinc from −20 to 280 °C with a scanning rate of 10 °C/min. About 10 mg of powder was crimped in aluminum pans and then exposed to a defined temperature program.

### Surface morphology of spray-dried powder

The surface properties of prepared particles were evaluated using scanning electron microscopy (XL30, The Netherlands). Particles of the corresponding samples were coated with gold at room temperature (BAL-TEC, Switzerland) and the applied accelerated voltage was set at 25 KV.

### Assessment of aerodynamic behavior of spray-dried powder

Deposition efficiency of prepared formulations was evaluated applying a twin-stage impinger (TSI; Apparatus A, European Pharmacopoeia, 2000, Copley, Nottingham, UK). About 10 mg of optimum-dried formulation, (42.38 mg, Cysteine; 122.72 mg, Trehalose; and 0.05 mg, Tween 20), was transferred into HPMC capsule size 2. The calibrated device in the mouthpiece was connected to the throat of the TSI. The pump was switched on a flow rate of 60 L/min for 5 s. The deposited powder in each section (inhaler, capsule shell, stages 1 and 2) was collected through rinsing with purified water and quantified by UV spectroscopy. The UV-absorbance of samples was detected at 280 nm. The RD was known as the total recovered amount of powder from all stages. The ED was defined as amount emitted from the inhalation device and capsule into the TSI. The amount of powder deposited in stage 2 of the TSI (effective cut-off diameter < 6.4 mm) was considered to be FPD. The ratio of FPD to RD was expressed as FPF.

### Optimization of experimental data

For determination of the optimized IgG formulations, (42.38 mg, Cysteine; 122.72 mg, Trehalose; and 0.05 mg, Tween 20), various 3-D response surface graphs were generated from the experimental data. The observed responses were fitted to linear and quadratic models and were evaluated to determine statistically significant coefficients and ρ values. Subsequently, the optimum values of the variables were provided by applying numerical and graphical analysis based on the desirable criteria of each variable. Optimized formulation was prepared and evaluated for different response characteristics. Finally, the achieved value of each response was quantitatively compared with that of predicted value for determination of corresponding predicted error.

## Results and discussion

The factors examined in this study were the amount of Cysteine (X_1_), Trehalose (X_2_), and Tween 20 (X_3_) as independent variables which were demonstrated by −1, 0, and +1 (Table 1). The evaluated dependent responses were yield (Y_1_), beta-sheet content (Y_2_), amount of aggregation at time 0 (Y_3_), and amount of aggregation following 2 months of storage at 45 °C (Y_4_) with determined constraints as described in Table 1. The experiment design matrix generated by the software is represented in Table 2.

Aqueous solutions containing antibody and excipients were spray-dried based on a Box–Behnken design under identical process conditions and then characterized with respect to yield of process, molecular stability of IgG within spray-drying, and after storage as well as secondary conformation of antibody.

The regression equations of the fitted models were plotted. The estimated regression coefficients given in Table 3 offer insights into the relationship between formulation components and reflect the relative significance of independent factors on the responses of the dependent variables. In all data analysis, probability values (*p*-value) less than 0.05 are considered to be statistically significant. The regression coefficients and *p*-value s for measured responses are listed in Table 3.

**Table 3:** Statistical analysis results of responses

Parameter	Response (Y1)		Response (Y2)		Response (Y3)		Response (Y4)	
	CE	*p*-value	CE	*p*-value	CE	*p*-value	CE	*p*-value
Intercept	37.42	< 0.0001	70.02	< 0.0001	0.14	< 0.0001	0.87	< 0.0001
X1	2.93	0.0015^a^	0.76	0.0153^a^	−0.1	0.0006^a^	−0.3	< 0.0001^a^
X2	2.81	0.0019^a^	3.16	< 0.0001^a^	−0.17	< 0.0001^a^	−0.57	< 0.0001^a^
X3	−0.19	0.7572	−0.1	0.7086	−0.13	0.0002^a^	−0.32	< 0.0001^a^
X1 × X2	1.97	0.0478^a^	–	–	0.03	0.2563	2.250E-004	0.8759
X1 × X3	1.98	0.0478^a^	–	–	−0.06	0.0427^a^	−0.14	0.0166^a^
X2 × X3	−1.30	0.1589	–	–	0.028	0.2945	0.092 0.	0858
X1^2^	−7.74	< 0.0001^a^	–	–	0.092	0.0061^a^	0.21	0.0022^a^
X2^2^	−4.26	0.0011^a^	–	–	0.29	< 0.0001^a^	0.73	< 0.0001^a^
X3^2^	0.99	0.2578	–	–	0.17	0.0002^a^	0.48	< 0.0001^a^

^a^ Significant factors with *p*-value <0.05

### Yield of spray-drying

A high powder yield is generally considered as the major requirement of the spray-drying process, especially for expensive proteins including antibodies [14]. The yield was quantified through dividing the amount of recovered powder to the total amount of initial solid content. The calculated yields ranged from 21.1 ± 0.2% to 40.2 ± 0.1% (Table 2). The following modified quadratic model was established for this response (R^2^ = 0.96 and *p*-value <0.0001):

2 $$ {\mathrm{Y}}_1=37.84+2.93\ {\mathrm{X}}_1+2.81\ {\mathrm{X}}_2+1.97\ {\mathrm{X}}_1{\mathrm{X}}_2+1.98\ {\mathrm{X}}_1{\mathrm{X}}_3\hbox{-} 7.68\ {{\mathrm{X}}_1}^2\hbox{-} 4.21\ {{\mathrm{X}}_2}^2 $$

**Fig. 1:**
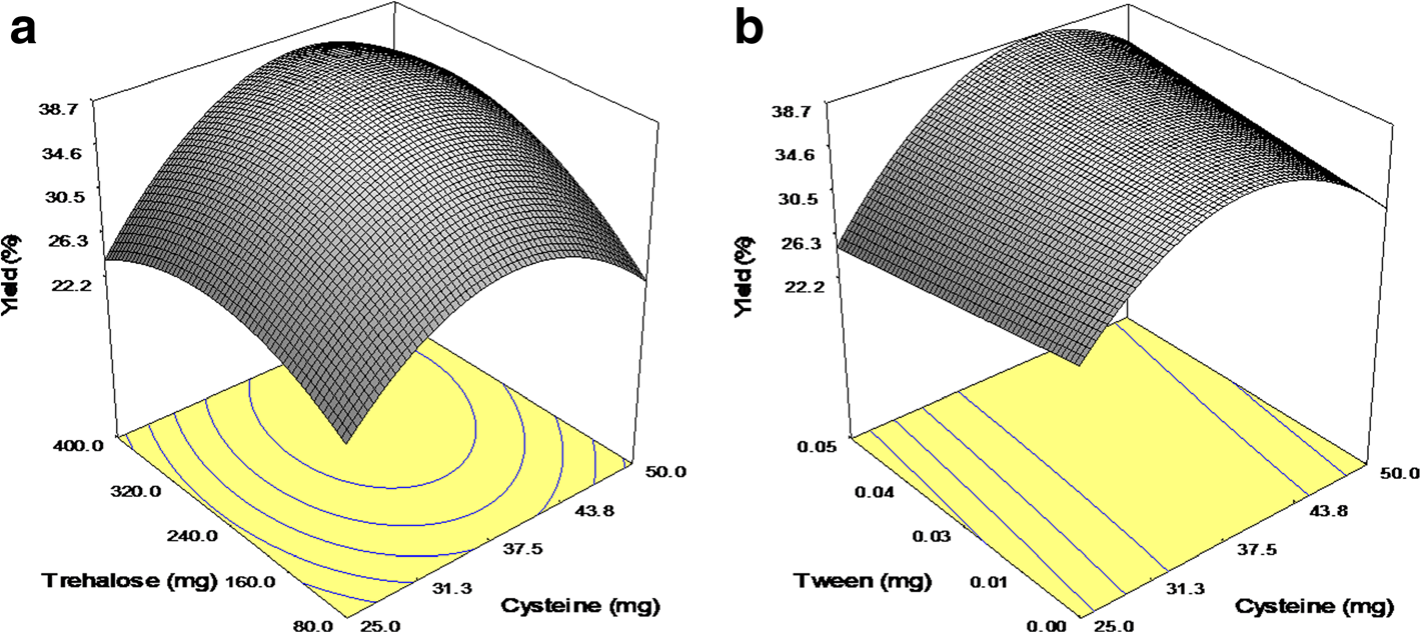
Response surface plot: effect of the additives on yield of process (*Y*_1_). **a** the amount of Cysteine (X_1_) and Trehalose (X_2_). **b** the amount of Cysteine (X_1_) and Tween 20 (X_3_)

It is evident that Cysteine and Trehalose positively affect the measured yield of process, thus showing proper interaction. Tween 20 was shown to insert no meaningful effect on the yield, but its interaction with Cysteine was regarded to be significant. The 3-D surface plots of interactions are exhibited in Fig. 1a and b. Incorporation of Tween 20 in to IgG solution prior to spray-drying resulted in the formation of sticky particles which adhere to the inner wall of the cyclone. Our finding was in accordance with the result of study performed by Adler et al. [15]. Positive interaction between Trehalose and Tween 20 with Cysteine could be due to hydrophobic nature of Cysteine. Coverage of the particles surface with Cysteine during spray drying prevented their adhesion to the cyclone and enhanced the yield.

### Secondary conformation of prepared powders

**Fig. 2:**
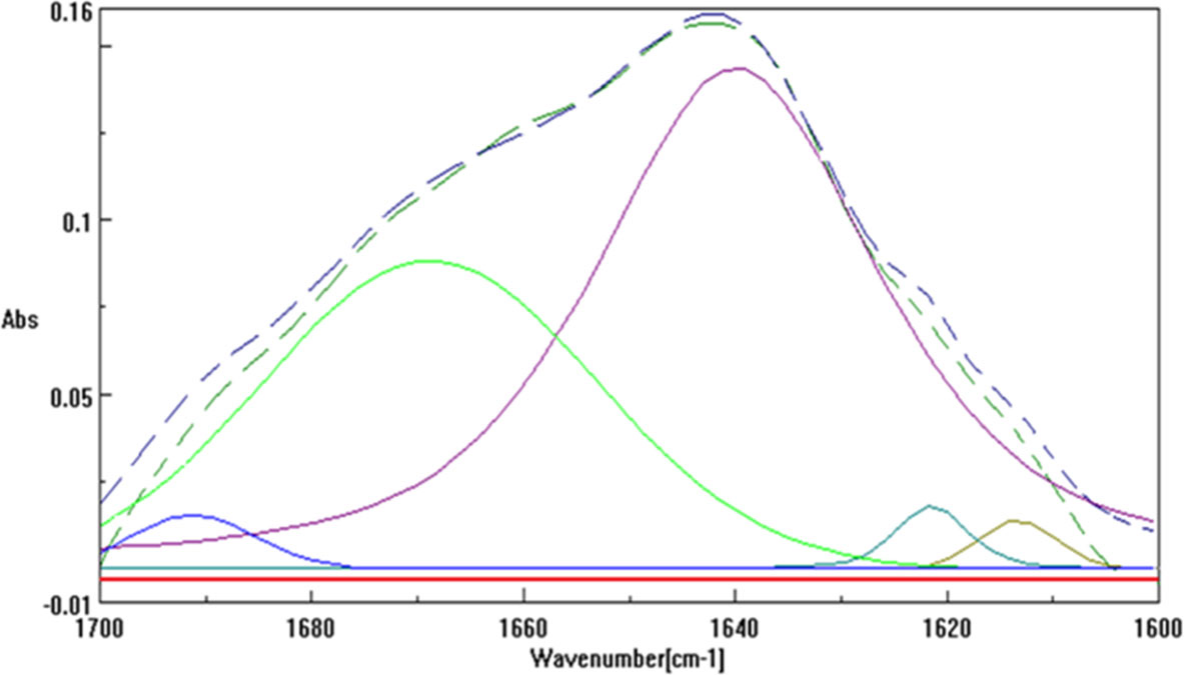
FTIR-spectra of spray-dried pure IgG: The original and fitted trace spectra (dashed lines), the resulted fitted- curves (solid lines)

FTIR spectroscopy is one of the most extensively applicable methods for studying structural changes in proteins upon spray-drying [16, 17]. The frequency of the Amid I band is in the range between 1600 and 1700 cm^−1^. The secondary structure of IgG is dominated by βeta sheet structures in 1614, 1639, and 1690 cm^−1^ (Fig. 2). Spectrum analysis with mixed Gaussian/Lorentzian fitted carves in 1600 to 1700 cm^−1^ revealed that the βeta sheet content in prepared formulations varied between 66.22 ± 0.19% to 73.78 ± 0.26%. Therefore, it is likely that in all the prepared formulations antibody structure was well conserved following spray-drying. The best fitted model for this response was linear (R^2^ = 0.92 and *p*-value <0.0001) as follows:

3 $$ {\mathrm{Y}}_2=70.02+0.76\ {\mathrm{X}}_1+3.16\ {\mathrm{X}}_2 $$

It is observable that the content of βeta sheet was significantly influenced by the presence of Cysteine and Trehalose with CEs of 0.76 and 3.16 respectively. As expected, Trehalose inserted the most stabilizing effect on protection and/or increasing the βeta sheet, the hydrogen bonds between the protein and water which were lost upon spray-drying [18, 19]. Contrary to Trehalose and Cysteine, the impact of Tween 20 on protecting the secondary structure of spray-dried IgG was statistically negligible (p-value: 0.2127). Therefore, the related term (X_3_) was eliminated from the final equation. Similarly, addition of Tween 20 to Anti-L-Selectin demonstrated no thermodynamic stabilization of antibody conformation [20]. Surfactants prevented protein adsorption at various interfaces. They can also interact with hydrophobic segments of the proteins [21]. In contrast to carbohydrates, surfactants have insufficient capacity to inhibit protein unfolding in the absence of water. Similar report was observed in the presence of Tween 20 which could not suppress unfolding of factor XIII [22].

### SEC- HPLC analysis

Antibody stability studies were performed on the amount of aggregation and/or fragmentation produced both immediately after process and on following accelerated storage conditions (temperature of 45 °C for 2 months).

### Molecular stability after spray-drying

Because the formation of soluble aggregates was severally reported to be the major degradation pathway of various antibodies, the physical stability of IgG was examined regarding the formation of soluble aggregates [23, 24]. The percentage of calculated aggregates varied from 0.11 ± 0.05% to 0.95 ± 0.3% for various factor level combinations after process (Table 2). A modified quadratic model was well-fitted for the physical stability of spray-dried IgG (R^2^ = 0.98 and *p*-value <0.0001), as demonstrated below:

4 $$ {\mathrm{Y}}_3=0.14\hbox{-} 0.1{\mathrm{X}}_1\hbox{-} 0.17{\mathrm{X}}_2\hbox{-} 0.13{\mathrm{X}}_3\hbox{-} 0.06{\mathrm{X}}_1{\mathrm{X}}_3+0.092{{\mathrm{X}}_1}^2+0.29{{\mathrm{X}}_2}^2+0.17{{\mathrm{X}}_3}^2 $$

As it was indicated, Cysteine, Trehalose, and Tween 20 significantly decreased the aggregation within this process. Trehalose inserted the most dramatic effect (CE of −0.17), on the physical stability of IgG, which is due to the formation of hydrogen bonds with the antibody in the absence of water. Carbohydrates like Sucrose and Trehalose are considered as the most applicable stabilizers in biological formulations. Among different carbohydrates, Ttrehalose is a unique one with the ability to form potent hydrogen bonds with protein molecules in the dried-state [25]. As a non-reducing sugar with high hydration capacity, Trehalose was shown to stabilize dried formulation of antibodies from both physical and thermodynamic aspects [18].

**Fig. 3:**
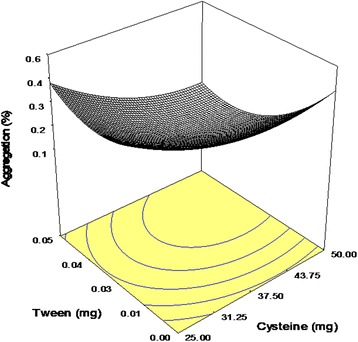
Response surface plot: effect of Cysteine (X_1_) and Tween 20 (X_3_) on the amount of aggregation following process (Y_3_)

**Fig. 4:**
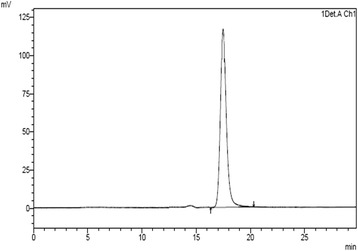
SEC- HPLC chromatogram of F_1_ containing Cysteine: 37.5 mg, Trehalose: 105 mg and Tween 20: 0.03 mg

Tween 20 and Cysteine were also influential additives demonstrating positive interaction with CE of −0.06, (Fig. 3). Polysorbates as non-ionic surfactants were repeatedly applied as protein stabilizers. Tween 20 contained fatty acid chains (as the hydrophobic part) and ethylene oxide segments (as the hydrophilic structure) [26, 27]. Surfactants are able to compete with proteins for denaturing interfaces [28], thus avoiding the exposure of protein to the spray-drying stress factors. Additionally, the presence of a hydrophobic part in the surfactant increases its ability to target the hydrophobic region of the unfolded antibody and protect it from self-association and intermolecular aggregation. The existence of synergistic effect between Cysteine and Tween 20 (with statistically significant interaction between Cysteine and Tween 20) might be due to further coverage of hydrophobic regions in antibody molecules, thus decreasing the aggregation more influentially. It is worth noting that in all prepared formulations, no detectable fragments were observed as a measurable peak after that for the IgG monomer (Fig.4).

### Molecular stability following 2 months of storage at 45 °C

Accelerated storage stability test at high temperature (45 °C) and RH of 60% was performed to further evaluate the impact of formulation components on protein stability. SEC-HPLC was carried out following 2 months to measure the percent of induced aggregates/fragments. The range of aggregates was varied from 0.8 1 ± 0.01% to 3.13 ± 0.64% (Table 2). A modified quadratic model was successfully fitted for the storage stability of spray-dried IgG (R^2^ = 0.99 and *p*-value <0.0001) as follows:

5 $$ {\mathrm{Y}}_4=0.87\hbox{-} 0.3{\mathrm{X}}_1\hbox{-} 0.57\ {\mathrm{X}}_2\hbox{-} 0.32{\mathrm{X}}_3\hbox{-} 0.14{\mathrm{X}}_1{\mathrm{X}}_3+0.21{{\mathrm{X}}_1}^2+0.73{{\mathrm{X}}_2}^2+0.48{{\mathrm{X}}_3}^2 $$

**Fig. 5:**
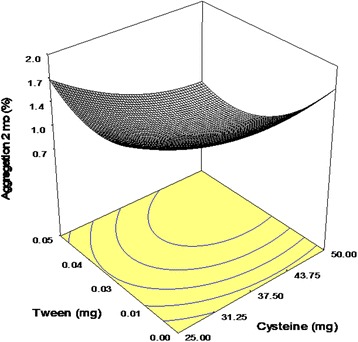
Response surface plot: effect of Cysteine (X_1_) and Tween 20 (X_3_) on the amount of aggregation upon storage (*Y*4)

Similar to short-term stability, no fragments were observed in the SEC-chromatogram of spray-dried powders after storage. All excipients significantly reduced the amount of soluble aggregate with positive interaction between Cysteine and Tween 20 (Fig. 5). Although polysorbates can stabilize proteins against surface denaturation within spray-drying, storage stability of prepared powders deteriorated in the presence of these surfactants [21, 29]. Auto-oxidation of several existing polyoxy ethylene groups in Tweens at high temperatures is considered as the major adverse effect of these additives, which could conclusively facilitate protein unfolding and aggregation up on accelerated storage conditions or induced protein fragments [12]. Since Cysteine has exhibited antioxidant activity in formulations of different peptides and proteins [30, 31], the improved synergistic effect between Cysteine and Tween 20 would be likely due to inhibition of Tween 20 auto-oxidation and the subsequent stabilization of the antibody. Generated peroxidase in polysorbates could cause degradation of proteins. The findings of performed examinations comparing effectiveness of various antioxidants concluded Cysteines as one of the best anti-oxidants in this regard [32].

### Optimization

Effect of independent variables on the responses was evaluated. The optimum levels of these variables were determined based on established constraints using a mathematical approach. The optimum formulation was prepared applying 42.38 mg Cysteine, 122.72 mg Trehalose and 0.05 mg Tween 20. The predictive values of each response, observed responses, and the predicted error are presented in Table 4.

**Table 4:** Comparative values of predicted and observed responses for optimized-IgG formulation (42.38 mg, Cysteine; 122.72 mg, Trehalose; and 0.05 mg, Tween 20)

Dependent variables	Predicted response	Observed response	Predicted error (%)
Y1	39.3134	38.5223	−2.0122
Y2	71.5596	70.8254	−1.0259
Y3	0.1099	0.1068	−2.8207
Y4	0.7616	0.7732	+1.5231

The results obtained from optimum formulation of IgG were in close agreement with the predicted values within 5% of the predicted error. The optimized formulation was subsequently characterized regarding thermal feature (by DSC analysis), particle morphology (through SEM) and aerodynamic behavior (with TSI).

### Thermal behavior of spray-dried powder

**Fig. 6:**
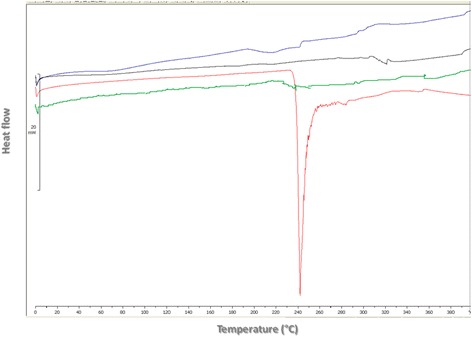
DSC- thermogram of spray- dried powders: spray-dried pure IgG (black peak), spray-dried Cysteine (blue peak), spray-dried Trehalose (red peak) and optimum formulation, (42.38 mg, Cysteine; 122.72 mg, Trehalose; and 0.05 mg, Tween 20), (green peak)

The thermal behavior of the optimum formulation was characterized applying DSC analysis (Fig. 6). Thermogram of spray-dried pure IgG (black peak) exhibited the amorphous nature of the pure antibody upon spray-drying. This finding was consistent with previous studies, which demonstrated that spray-dried IgG remained fully amorphous [33]. Similarly, the absence of significant endotherms in thermograms of spray-dried Cysteine (blue peak), spray-dried Trehalose (red peak), and optimum formulation (green peak) was attributed to amorphous nature of these compounds following spray-drying.

### Surface morphology and aerodynamic performance of spray-dried powder

**Fig. 7:**
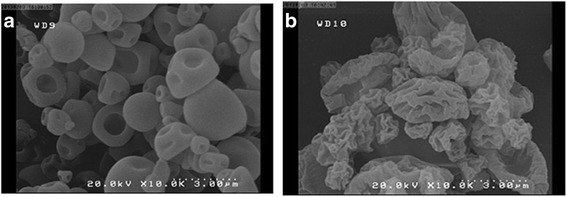
SEM- photograph of spray-dried powders. **a** Pure- IgG, **b** Optimum formulation (42.38 mg, Cysteine; 122.72 mg, Trehalose; and 0.05 mg, Tween 20)

The surface morphology of the optimum formulation was compared with that of the pure antibody by applying scanning electron microscopy (Fig. 7a and b). Donut-like particles with smooth surfaces were created through spray-drying pure antibody, which was in total agreement by other performed studies [34]. Based on our previous study, the addition of Cysteine caused the formation of raisin-like particles with enhanced aerodynamic behavior [13]. In the current investigation, the optimum formulation of IgG in the presence of Cysteine, Trehalose, and Tween 20 similarly demonstrated the generation of highly corrugated and raisin-like particles which could be appropriated for aerosol delivery of spray-dried antibody with desirable FPF and ED values of 68.75 ± 0.79% and 93.56 ± 1.12% respectively.

## Conclusion

A Box-Behnken experimental design successfully helped in understanding the interaction effects between the three applied additives. The stability and particle properties of spray-dried IgG improved significantly in the presence of Trehalose, Tween 20, and Cysteine. This is in keeping with our previously developed formulation carried without Tween 20. Tween 20 oxidation at elevated temperatures can yield reactive peroxides that will attack the antibody, causing fragmentation as well as aggregation which cannot be observed in the presence of Cysteine. Further work exploring the assumption that Cysteine on Tween auto-oxidation has an anti-oxidant effect will be reported in our future publications.

## Abbreviations

CE: Coefficient estimate
DSC: Differential scanning calorimeter
ED: Emitted dose
FPD: Fine particle dose
FPF: Fine particle fraction
FTIR: Fourier transformi nfra- red
IgG: Immunoglobulin G
KBr: Potassium bromide
KDa: Kilo dalton
Mg: Milligram
RD: Recovered dose
RH: Relative humidity
SEC-HPLC: Size exclusion chromatography – high pressure liquid chromatography
SEM: Scanning electron microscopy
